# Supplementary material for: Patterns of Intron Gain and Loss in Fungi
Source: PLoS Biol. 2004 Nov 30;2(12):e422. doi: 10.1371/journal.pbio.0020422 (PMC532390; doi:10.1371/journal.pbio.0020422)
Supplement: Table S1 — Also available at http://genes.mit.edu/NielsenEtAl/. (4.3 MB ZIP). [file pbio.0020422.st001.zip › NielsenEtAl/html/511.html]

AN0034.1.NCU03779.1.MG04014.1.FG07017.1


```
 CLUSTAL W (1.82) Multiple Sequence Alignments - Introns Inserted


Sequence 1: NCU03779.1	649 aa
Sequence 2: FG07017.1	579 aa
Sequence 3: MG04014.1	593 aa
Sequence 4: AN0034.1	590 aa
Alignment Length: 656 aa
Number Identitical Residues: 264 aa
Alignment Score (without introns) 12582


MG04014.1 	-MSA~RHFVNDPTHLVNTALHAITITNPSVACDAENKIIYVRP---------DSRRPRQP
NCU03779.1	-MSN~RHFINDPQHLVLTSLQSTTLLNPGIALDAHNKIVYLRPGYGNASSSSSSSNQRQK
FG07017.1 	-MSA~KHFVNDPTHLVSSALHSLTLTNPSVALDPEFKVIYRRP----------DSNVKPQ
AN0034.1  	MAQT1KHFFSDPTHLVHTALNSLTLTNPSLAFDRENKIIFRRP----------DVVRKGK
          	  .  :**..** *** ::*:: *: **.:* * . *::: **          .   :  

MG04014.1 	VAIISGGGSGHEPSFGGMVGRGLLSAAVAGTIFASPSTEQVRTAITSRVEVGDR------
NCU03779.1	VSLISGGGSGHEPSFSSMVGRGLLSACVAGTIFASPSAEQVRTAIMGRVENNEPSSSPEH
FG07017.1 	VSIISGGGSGHEPSFAGMVGQGMLSAAVAGTIFASPSAEQIRAAITSRVDTSK-------
AN0034.1  	VAIISGGGSGHEPAFAGFVGQGLLDASAAGTIFASPNAEQIRIAAMERVNNEQ-------
          	*::**********:*..:**:*:*.*..********.:**:* *   **:  .       

MG04014.1 	-------ADGGGVLITVMNYTGDILNFGVAAEKARAAGINVEMVVIGDDVGVGRAKGGKV
NCU03779.1	GQQQQRGEDAGGVLVVVMNYTGDVLNFGLAVEKAKAAGVNVEMVVVGDDVGVGRAKAGKV
FG07017.1 	-----------GVLVTVMNYTGDVLNFGMAVEKGKAAGLDVEMVVVGDDVGVGRAKAGKV
AN0034.1  	-----------GVLIIPMNYTGDVLNFGMAAEKSRAAGIKTEFFAINDDAGVGKTKGGKV
          	           ***:  ******:****:*.**.:***:..*:..:.**.***::*.***

MG04014.1 	GRRGIAGTVLVDKIAGALAARG~ANLSEVTHVARLVASNVASIGASLEHVHVPGRKAADQ
NCU03779.1	GRRGIAGTVLVQKIAGALAAQG~RSLKEVTKVANLAAENIVSVGASLEHVHVPGRRAEAE
FG07017.1 	GRRGIAGTVLVHKISGALAALG~KPLEQVAKYAQLTADNLVSVGASLEHVHVPGRKVDTE
AN0034.1  	GRRGIGGGVLILKIVGALAEAG2GSLEEVYKTAQLANENLASVGSSLEHVHVPGREPSDD
          	*****.* **: ** ****  *   *.:* : *.*. .*:.*:*:**********.   :

MG04014.1 	ED---TLAEGEVEIGMGIHNEPGSGREKDLELPALVERMLAQLLDQKDADRAYTQVGSGD
NCU03779.1	GAEFKVLKEGEVEIGMGIHNEQGSSREKGIGLPELVGKMLKQLLDRNDEDRAFVNVNS-N
FG07017.1 	AS----LAADEVELGMGIHNEPGSGREK-VELPDLVTKMLQQLLDANDKDRAFVNVSS-K
AN0034.1  	HIP-----EGEVEIGMGIHNEPGSTRTK-TTLVDLVATMLLQILDHNDPDRSYITHSPGD
          	         .***:******* ** * *   *  **  ** *:** :* **::   ....

MG04014.1 	KFVLLVNNLGGVSVLEMGGITAEVAKQLGTT-WAIKPVRVLAGTFMTSLNGLGFSISLLK
NCU03779.1	EVVLLVNNLGGVSPLEMGGIVTEVVTQLAEQPYGIKPVRILSGTYMTSLNGLGFSISLLN
FG07017.1 	QVILMINNLGGVSVLEMGGITAEVASQLESK-YDIHPVRVLSGTYMTSLNGLGFSISLLN
AN0034.1  	KFVLLVNNLGGLSTLELSGITDEVYRQLGKS-YQIKPERVIQGTFLTSLNGLGFSISLLK
          	:.:*::*****:* **:.**. **  **    : *:* *:: **::*************:

MG04014.1 	VVDTGS-SAT-MVQLLDDPCEATGWSAPIATETWDSP--PTATKEGNLKEERDLKPTGLR
NCU03779.1	VVNTDI-GGPGMIDLLDYPSEATGWTAPISKLTWEEQRENKSTRENEVKSEEEVRPCGLT
FG07017.1 	VVAPDF-DAPSMIELLDTPSEVVGWSAPIKTSTWEAK--NTSTRTDRVSATEDIKSSDLK
AN0034.1  	LADTGLGPGKSFLELLDAPAEAVGWSAPIKPATWEYR--NAPGIEVKRAKPAEQPPSNVK
          	:. .. . . .:::*** *.*..**:***   **:      .    .     :  . .: 

MG04014.1 	L~DAAVAQNAITAALENVIAVEPEVTKYDTVVGDGDCGIGLRRGAEA1VLKHLA--SNPL
NCU03779.1	Y~EPQEVQKQLVAGLKAVIAAEPDVTKYDTMVGDGDCGIGLKRGAEA1ILSHLD--SNPL
FG07017.1 	T~DASSAQSALKNALQKVIDAEPEVTRYDTVVGDGDCGIGLKRGAEA1ILKHME--QRSL
AN0034.1  	L1DIAKVRKVLGAALKRMIDAEPQITRYDTIVGDGDCGVGLKRGAQA~VLDLLNDASANL
          	  :   .:. :  .*: :* .**::*:***:*******:**:***:* :*. : .:.  *

MG04014.1 	SGDAAVDVASIVPVVENHMDGTSGALYSIFLNAMLRSLRDNAPSSGES-TASPELWAKVL
NCU03779.1	TGDAAIDLAKIVTVVEQTMDGTSGALYGIFLNALVASLHTVGGGESDKKAADENAWAAAL
FG07017.1 	TGDVVVDLARIVTIVETAMDGTSGALYAIFLNALVKALRELAPGN-----ASPEVWGKAL
AN0034.1  	NDDIVHTVNRIVTVVENTMDGTSGAIYAIFLNALVHGLREQDKGTETP--ADTDVWGTAL
          	..* .  :  **.:**  *******:*.*****:: .*:    .      *. : *. .*

MG04014.1 	KESCKALGNYSQAQVGDRTLIDALHPFVNTLAETGNVGTAASAAQKGAESTKGMKPSFGR
NCU03779.1	KLSCEALSKYTPARPGDRTLVDALYPFVDALEKTGDVKKAAEAAKQGAEGTKGMKAKLGR
FG07017.1 	RKSSDALARYTPARPGDRTLVDALHPFVEVLNQTGDVKKAAEAALEGANKTKGMQASLGR
AN0034.1  	KYSISALGKYTPAQVGDRTMIDALVPFAQTLADKRDVHAAAKAAEEGTEATKHMKASLGR
          	: * .**..*: *: ****::*** **.:.* .. :*  **.** :*:: ** *:..:**

MG04014.1 	STYVGGTG--FEQVPDAGAWGLAAFFNGLARQS---------------------------
NCU03779.1	TVYIGGSG--FEQVPDPGAWGLAKFFLGLAGIEKADDSSAKQAKGSDDGWEEVTREAELP
FG07017.1 	TVYIGGGG--YQEVPDPGAWGLACFFLGLAG-----------------------------
AN0034.1  	AVYVGGEQEWVGKVPDPGAYGLSEFFTGLAGAL---------------------------
          	:.*:**  .   :***.**:**: ** ***                              

NCU03779.1	
FG07017.1 	
MG04014.1 	
AN0034.1  	
          	
```
